# Quantifying Water Flow within Aquatic Ecosystems Using Load Cell Sensors: A Profile of Currents Experienced by Coral Reef Organisms around Lizard Island, Great Barrier Reef, Australia

**DOI:** 10.1371/journal.pone.0083240

**Published:** 2014-01-08

**Authors:** Jacob L. Johansen

**Affiliations:** ARC Centre of Excellence for Coral Reef Studies, and School of Marine and Tropical Biology, James Cook University, Townsville, Queensland, Australia; Macquarie University, Australia

## Abstract

Current velocity in aquatic environments has major implications for the diversity, abundance and ecology of aquatic organisms, but quantifying these currents has proven difficult. This study utilises a simple and inexpensive instrument (<$150) to provide a detailed current velocity profile of the coral-reef system around Lizard Island (Great Barrier Reef, Australia) at a spatial and temporal scale relevant to the ecology of individual benthos and fish. The instrument uses load-cell sensors to provide a correlation between sensor output and ambient current velocity of 99%. Each instrument is able to continuously record current velocities to >500 cms^−1^ and wave frequency to >100 Hz over several weeks. Sensor data are registered and processed at 16 MHz and 10 bit resolution, with a measuring precision of 0.06±0.04%, and accuracy of 0.51±0.65% (mean ±S.D.). Each instrument is also pressure rated to 120 m and shear stresses ≤20 kNm^−2^ allowing deployment in harsh environments.

The instrument was deployed across 27 coral reef sites covering the crest (3 m), mid-slope (6 m) and deep-slope (9 m depth) of habitats directly exposed, oblique or sheltered from prevailing winds. Measurements demonstrate that currents over the reef slope and crest varies immensely depending on depth and exposure: Currents differ up to 9-fold within habitats only separated by 3 m depth and 15-fold between exposed, oblique and sheltered habitats. Comparisons to ambient weather conditions reveal that currents around Lizard Island are largely wind driven. Zero to 22.5 knot winds correspond directly to currents of 0 to >82 cms^−1^, while tidal currents rarely exceed 5.5 cms^−1^. Rather, current velocity increases exponentially as a function of wave height (0 to 1.6 m) and frequency (0.54 to 0.20 Hz), emphasizing the enormous effect of wind and waves on organisms in these shallow coral reef habitats.

## Introduction

Current is a fundamental feature of aquatic ecosystems and has implications for all aspects of aquatic life [1]–[4]. Variation in current velocity can shape the relative distribution and abundance of numerous species and families of aquatic organisms [5]–[6], and directly affect settlement, growth and survival of individuals [7]. While many important ecological patterns have been attributed to the level of currents within marine, brackish and freshwater ecosystems [5], [7]–[10], few studies have directly quantified these currents over the spatial and temporal scales that affect individuals and populations.

A plethora of current sensors is currently being used by oceanographers worldwide including Electromagnetic Current Meters and Acoustic Doppler Current Profilers (ADCP) [11]. These instruments allow long term monitoring for months to years and some are capable of high resolution profiling of the entire water column around each instrument including near bottom velocity measures [12]–[14]. However, instruments such as the ADCP's are very expensive ($10,000–25,000 per instrument) and in most cases not economically viable for profiling currents in multiple locations simultaneously. The high cost of the commercially available sensors have so far impede current related studies in many fields other than physical oceanography and forced researchers to choose between long-term measurements at few points and high spatial resolution mapping of the current regime [15]–[16].

Ecological studies that require measures of current velocity have often resorted to low cost plaster dissolution methods or spring-type dynamometers [5], [8], [17]–[18]. Plaster dissolution correlate ambient current velocity with the rate of dissolution under different current regimes. While relatively inexpensive and can be deployed in multiple locations to increase the spatial scale of measurement, this method only provides estimates of bulk volume flux over the entire period of deployment. Ecologically, however, maximum current velocities and variations in velocity are of greater importance than average velocities since habitat use by many aquatic organisms is limited by the strongest commonly occurring currents [5], [19]–[20]. These currents impose the greatest physical and physiological demands on resident species, and may directly dislodge sedentary organisms and reduce foraging ability [8]–[9], [21]. Consequently, ecologically meaningful measures of current must include maximum velocities encountered within the spatial scales of the individual as well as the duration and frequency of these maximum velocities.

Spring-type dynamometers can produce low resolution measures of maximum current velocities in an environment [17]. However, each instrument is only capable of recording a single value during deployment. To tease out the duration and frequency of these maximum forces, continuous measures are necessary. Tilt sensors embedded within floats tethered to the bottom have been used with some success for continuous measures [22]–[23], however these designs often suffer from low resolution particularly at higher current velocities. Donelan and Motycka [24] proposed to use a strain gauge instrument to provide high resolution and continuous measures of currents based on drag forces exerted on a sphere. Although the concept was proven to work [25], this instrument had high power requirements and no internal power or data storage capacity. Lack of instrument autonomy limited deployment to areas and conditions where power source and data could be maintained at a surface station, and severely reduced the number of instruments which could be deployed simultaneously [26]. A recent study by Mach et al [27] demonstrated that autonomy can be achieved in this type of instrument, although build cost remained high. To elucidate the importance of currents for organism ecology and the conditions under which individual aquatic organisms survive, there is a great need for an automated but also practical, inexpensive and easily applicable method that will allow a greater number of researchers to conduct continuous and detailed profiling of currents in environments such as rivers, estuaries and coral reefs.

This study uses of a low power and inexpensive strain gauge instrument to produce a detailed current velocity profile across depth and exposure gradients of a tropical coral reef ecosystem, and thereby help cover a significant gap in our knowledge of the currents encountered by individual benthos and fishes on coral reefs. Each instrument costs <$150 in materials and is capable of obtaining continuous, accurate and precise measures of maximum current velocities and variations in velocities over time scales ranging from seconds to months

## Materials and Procedures

### Current meter construction

An instrument for continuously measuring and logging current velocity was developed in June 2009 using load cell technology commonly used by industry as a simple and reliable measure of force. A load cell sensor is simply a metal alloy force transducer which produces an analog output signal proportional to the applied weight or force. As force increases on the cell, the internal resistance increases causing a change in output voltage. These sensors require little power, provide linearly increasing output voltage with increasing force and are capable of retaining high precision (<0.01%) and high accuracy (<0.01%) for extended periods (see Table 1 for load cell performance characteristics).

**Table 1:** Load cell performance characteristics.

Load cell terminology	*Recommended range*	*Definition*
*Creep*	±<0.01%/hr	Creep is the change in output occurring over time while under load with all environmental conditions and other variables remaining constant. Creep is due to thermoelastic effects, i.e. the adiabatic heating and cooling of elastic load supporting elements within a load cell as they undergo deflection in response to changes in the applied force [39].
*Creep recovery*	0.1% of R.O./20 min	The change in rated no-load output (R.O.) occurring with time after removal of a load which had been applied for a specific period of time.
*Drift*	±<0.02% hr^−1^	A random change in output under constant load conditions.
*Error*	±0.01%	The algebraic difference between the indicated and true value of the load being measured.
*Hysteresis*	±0.01%	The maximum difference between load cell output readings for the same applied load.
*Nonlinearity*	±0.01%	The maximum deviation of the calibration curve from a straight line drawn between the no-load and rated outputs.
*Repeatability*	±0.01%	The maximum difference between load cell output readings for repeated loadings under identical loading and environmental conditions.
*Resolution*	>1024 divisions	The smallest change in mechanical input which produces a change in the output signal.
*Response time*	<5 ms	The time it takes the load cell to stop oscillating and settle at a reading. A quick return-to-zero is needed for areas of highly oscillating currents (e.g. high wave exposure)
*Temperature effect on rated output*	±<0.002%/°C	The change in rated output due to a change in ambient temperature.

Load cells must be chosen to fit the conditions where the instrument will be used. Although creep is unavoidable in most high precisions sensors, the effect can be accounted for by choosing a low creep load cell and including zero measures in the logged data for continuous zero verification and adjustment. Zero creep affects bias only and has no effect on the scale factor ensuring measurement stability during long term deployment.

**Figure 1:**
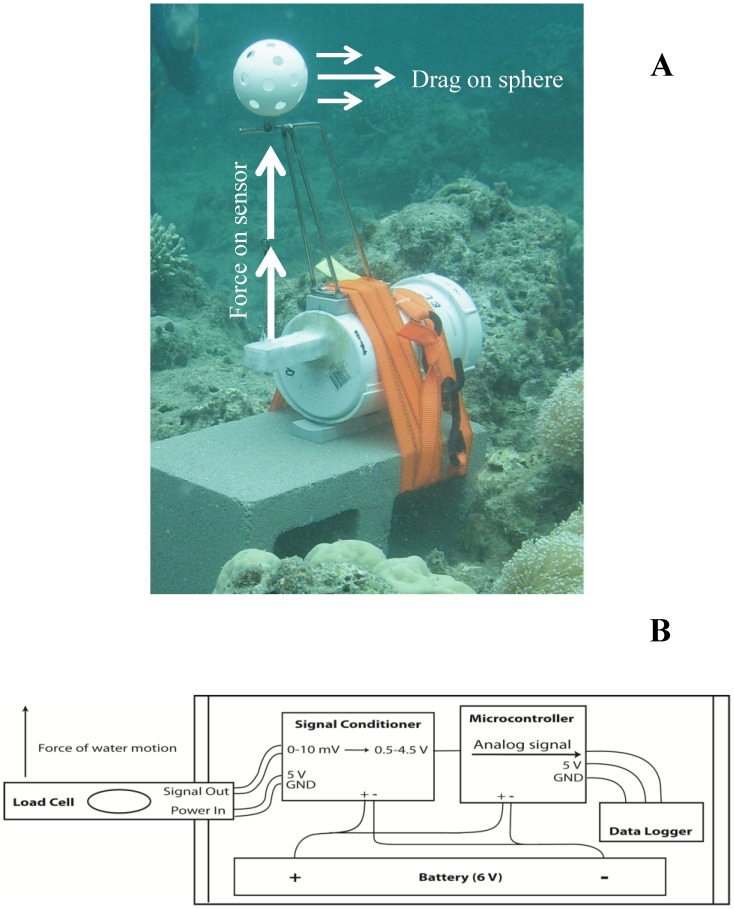
A picture and a diagram of current meter components. Top **A** shows an instrument deployed on a sheltered reef location with clear depictions of the guide rod construction and drag-sphere placement. Bottom **B** illustrates the internal components of the current meter and wiring connections. Notice that the height above the substratum where current measures are conducted can be adjusted by increasing or decreasing the guide rod length.

A single point load cell sensor (model 628A from www.hzloadcell.com) capable of measuring ≤3 kg loads with 0.015% precision was chosen for this experiment. This sensor has a natural resonance frequency of ∼500 Hz, allowing signals to be recorded to >100 Hz without incurring significant error due to resonance. The sensor was coated with a thin layer of silicon (i.e. submersible) and securely fastened to a submersible instrument housing (PVC pipe: diameter: 11 cm; length: 20 cm; pressure rated to 120 m water depth) with only the force measuring end of the cell protruding horizontally out of the housing (see schematics in Fig. 1 as well as component list and assembly instructions in Fig. S1 and Table S1). Single point load cell sensors measure force in one end only whilst the other end acts as an anchoring point. These sensors require all exertion forces to be perpendicular to the surface of the sensor. A stainless steel frame was therefore attached to the outside of the instrument housing with a 10 mm stainless steel guide rod positioned 30 cm directly above the sensor (see photo in Fig. 1). This guide rod can be placed at any desired height above the sensor, allowing the instrument to be tailored to suit a variety of organisms by measuring current closer or farther from the substratum. A 0.5 mm zero stretch line was then attached to the sensor measuring point, passed through the guide rod, and attached to a neutrally buoyant 7 cm diameter drag-sphere (whiffle ball) approximately 0.5 cm above the guide rod (Fig. 1). As a result, currents that produced a drag on the sphere were instantaneously registered by the sensor below (see picture in Fig. 1).

The instrument housing was then fitted with a signal conditioner, a data logger and a battery pack. The signal conditioner was connected between the battery and the sensor and had two functions: 1) to ensure any change in voltage output from the sensor was due to the forces acting on the sensor rather than slight variations in the voltage supplied by the battery pack; and 2) to amplify the output voltage from the sensor to a signal recognized by the data logger. Numerous commercial signal conditioners exist: This instrument used an INA122P instrumentation amplifier from Texas Instruments (electrical circuits can be seen at http://www.ti.com/lit/ds/symlink/ina122.pdf, accessed May 2013)

The data logger (consisting of a microcontroller and logger) was connected directly to the signal conditioner, and allowed the analog signal coming from the load cell sensor to be registered and recorded. Because analog sensors send out continuous signals, measurements of current velocity can be conducted at any frequency required, depending on the level of detail needed and the type of signal conditioner and data logger used. This instrument used a programmable Arduino Pro 328 5 V/16 MHz microcontroller and a Sparkfun Openlog data logger (for schematics see www.sparkfun.com). This microcontroller registers analog signals from 0–5 V at 10 bit and relays signals at user determined frequencies ≤16 MHz to the data logger. The Openlog data logger then automatically logs every signal sent from the microcontroller to a micro SD-card at a rate of ≤1 MHz. The load cell sensor and data logger used for this setup had resolutions of 60,000 and 1,024, respectively, providing a total measuring resolution of 1,024 divisions between 0–100% drag on the sphere. All components were run by a 6 V 7.2 Ah sealed Acid-lead rechargeable battery. These batteries have little heat build-up and do not release gaseous fumes, making them suitable for usage in hermetically sealed instruments and under elevated atmospheric pressures. All components and the battery were fitted within the watertight PVC housing, which had an O-ring fitted screw cap access point (Fig. 1).

### Current meter calibration and measurement validation

Current meter instruments were calibrated in two ways before deployment. First, the recorded signal from each sensor was calibrated to ensure equal accuracy and precision between instruments under equal drag force: The output signal from the load-cell sensor was 0–10 mV and the data logger recorded signals from 0.5–4.5 V, which required the signal conditioner to amplify the load-cell signal by (4.5−0.5 V)/(0.01−0.00 V) = 400 times. Although the data logger could record signals from 0–5 V, this study controlled for potential drift in the signal over time by specifying 0–100% drag on the sensor as 0.5–4.5 V. The exact amplification of the signal may be slightly different between instruments due to minute manufacturing differences (e.g. wiring resistance), but provide accurate signals to the data logger at any load between 0–100% when adjusted correctly. Signal amplification of each instrument was therefore adjusted using a variable resistor by placing 100% load on the sensor (i.e. 3 kg for this load-cell). Additionally, although the sensor had a precision of 0.015%, additional error is expected in electrical circuits due to internal component voltages causing imprecise output signals. The instrument amplifier used here(INA 122P) created a maximum electrical noise in the amplified output signal of 0.28 mV. This precision error was corrected in each instrument using another variable resistor by placing 0% load on the sensor and offsetting the output signal to 0.5 V. Calibrations of amplification levels and offset, therefore, allowed equally accurate and precise sensor signals from 0–100% load on all instruments.

Following these calibrations, instrument performance was validated by testing the precision and accuracy of each instrument. Precision was calculated from the standard deviation of three repeated measures obtained by hanging precision weights off the sensor of 13, 27, 40, 53, 89 and 100% of max load (i.e. 3 kg). Accuracy was calculated as ((measured load – applied load)/applied load)×100.

Second, ambient water velocities were correlated with measures of drag on the sensor sphere: To increase accuracy of calibrations at low current velocities, the instruments were initially placed in a flume (29×40×360 cm: width×depth×length) and exposed to incrementally increasing current velocities between 0 and 50 cms^−1^ (resolution of 0.25 cms^−1^). The flume was calibrated using video recordings of neutrally buoyant particles drifting past a grid. For higher current velocities up to 400 cms^−1^ the instruments were placed in a unidirectional current immediately next to a Vernier LabQuest Flow rate sensor with a resolution of 0.5 cms^−1^ following the boat-tow methodology of Utter and Denny [28].

### Field application on a coral reef

**Figure 2:**
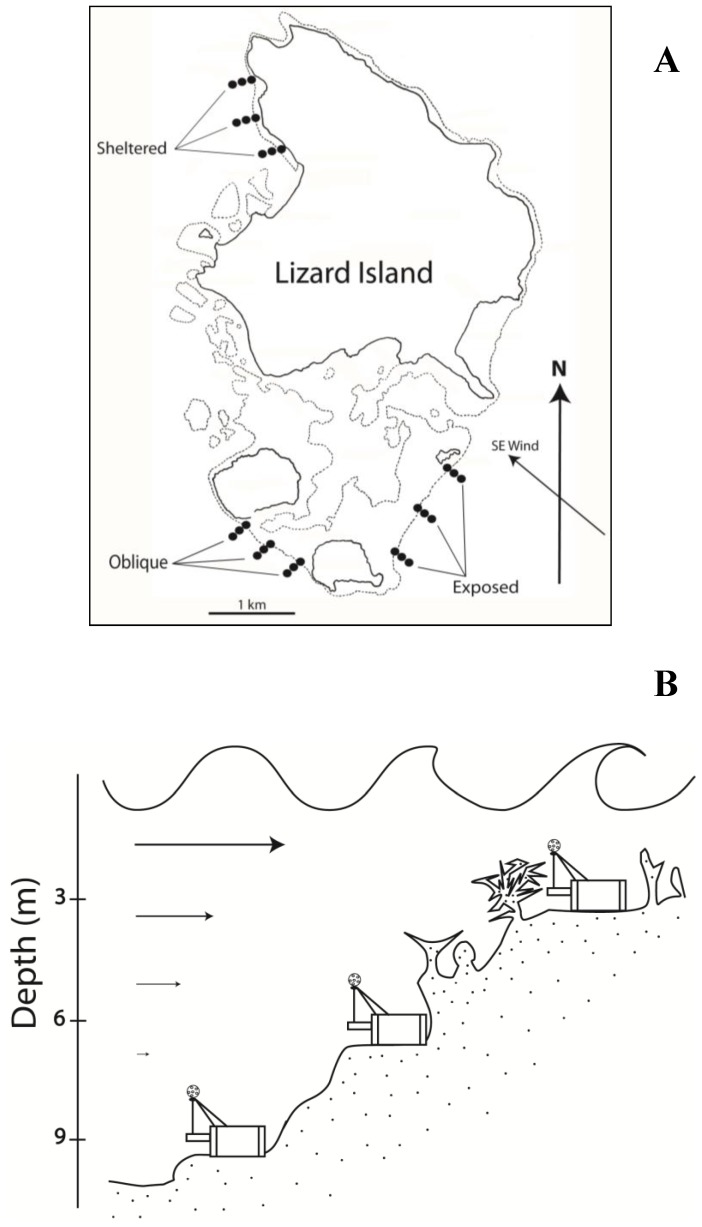
An illustration of measuring locations (**A**) and current meter placement (**B**) on each reef slope around Lizard Island, Northern Great Barrier Reef, Australia (14°40S, 145°28E). Current meters were placed at 3, 6, and 9 m depths at mid-tide in exposed, oblique and sheltered habitats relative to the prevailing south-easterly trade winds. These placements followed the approximate depths of the crest, mid and deep-slope of each habitat.

Instruments were then utilised to create a profile of wave and tidal-driven currents that was adequately detailed for ecological studies of individual benthos and fishes. The profile was created across a section of coral reef surrounding Lizard Island, Northern Great Barrier Reef (GBR), Qld, Australia (14°40S, 145°28E), with permission from the GBR Marine Parks Authority (permit G09/32235.1), during March, June and Feb 2010–2011, but could equally have been conducted in estuarine or river environments. This location is a shallow water mid-shelf coral reef directly exposed to the surrounding water basin but largely protected from open ocean swell (see also studies by Madin *et al.*, 2006 [8] and Fulton & Bellwood 2005 [5] for further details on waves and flow conditions across this reef environment). Twenty-seven sites were chosen for measures of current velocity and wave movement: three exposure habitats (“exposed”, “oblique” or “sheltered” reef relative to the prevailing south-easterly trade winds) were chosen and three locations within each habitat were sampled at three different depths. Locations within each habitat were separated by ∼300 m, and situated at 3, 6 and 9 m depth at mid tide following the crest, mid-slope and deep-slope of these reefs (Fig. 2). At every site, a current meter was secured to anchoring points in the substratum floor using ratchet straps. Each instrument was set to register changes in drag forces at 10 Hz, and recorded the time, date and the single highest point measure of current velocity every 10 seconds. Each instrument also recorded a series of 80 point measures at 10 Hz once every 60 seconds to allow calculations of wave frequency based on the wave driven pulses of drag on the drag-sphere. This equated to 8.800 drag measures per megabyte used, theoretically allowing the instruments to be deployed for over 35 days with a 500 mb SD-card. Current and wave data were recorded at wind velocities from 0 to 22.5 knots in each habitat, which corresponds to the range of weather conditions commonly encountered in this location (Australian Bureau of Meteorology and Australian Institute of Marine Science). A total of 2,258 hrs of current measures were recorded in the field, with an average of 251±47 hrs (mean ±S.E., range 161–528 hrs) in each habitat.

### Data analysis

Precision and accuracy was compared across instruments using a 2-way ANOVA with applied load and instrument as fixed factors. Data were square-root transformed to meet assumptions for ANOVA.

To account for the effect of winds and waves during field deployment, velocity measures were compared directly to concurrent weather conditions. Prior to September 2010, the most detailed weather data available for this location was hourly recordings of wind velocity, direction and wave height from the Australian Bureau of Meteorology [29]. Post September 2010, wind velocity and direction were available at 10 min intervals from the Australian Institute of Marine Science [30]. In both cases wind recordings reflected the average of the 10 min period prior to recording time (see Weather Station No 31213, www.bom.gov.au and Lizard Island Weather Station, http://data.aims.gov.au).

To avoid pseudo-replicating multiple current measures onto each individual weather recording, the analysis only used the single highest measure of current velocity and associated wave frequency of each concurrent weather recording. These highest measures were chosen because wave driven current velocities are highly variable and the ability of aquatic organisms to occupy a habitat is limited by the highest commonly occurring current velocities (e.g. [15]–[16], [20], [31]). In total, the 2,258 hrs of current measures provided 7,190 independent measures of current velocities concurrent with available weather data.

Due to high variation in current velocity within and among habitats, the distributions of drag measures were non-normal and could not be transformed to comply with assumptions of normality and homogeneity of variances necessary for linear models. Consequently, differences among locations of equal depth and exposure were examined using a series of Kruskall-Wallis ANOVA's (location as fixed factor). Where no difference in drag force was found among locations, data were pooled to represent nine distinct habitats: exposed, oblique and sheltered habitats at each of the three depths (3, 6 and 9 m, see Table 2). Differences among these nine habitats were then compared using Kruskall-Wallis ANOVA Multiple Comparisons test, followed by False Detection Rate (FDR) correction to avoid Type I errors [32]. Finally, field measures of drag forces in Newton (N) were converted to current velocities in cms^−1^ and plotted relative to wind velocity, whilst wave frequency was plotted relative to concurrent wave height estimates [30]. To account for time delay in wind driven wave propagation and current velocity [33]–[34] each velocity measure was correlated with the average wind over the preceding 3 hrs.

**Table 2:** Statistical comparison of current measures from 27 different sites on a coral reef spanning 3 levels of wind exposure (exposed, oblique and sheltered) and 3 different depths (3, 6 and 9 m).

Kruskall-Wallis ANOVA (by rank)															
A) Comparison of individual locations						B) Multiple comparisons among pooled habitats									
Habitat	Depth (m)	H	df	P	N	Habitat	Exp 3 m	Exp 6 m	Exp 9 m	Obl 3 m	Obl 6 m	Obl 9 m	Shl 3 m	Shl 6 m	Shl 9 m
Exposued	3	4.924	2	0.09	1659	Exp 3 m		<0.01	<0.01	<0.01	<0.01	<0.01	<0.01	<0.01	<0.01
	6	4.522	2	0.10	1523	Exp 6 m	27.86		<0.01	<0.01	<0.01	<0.01	<0.01	<0.01	<0.01
	9	2.686	2	0.26	717	Exp 9 m	32.37	10.11		<0.01	**1.00**	<0.01	**1.00**	<0.01	<0.01
Oblique	3	0.394	2	0.82	299	Obl 3 m	9.15	6.54	12.67		<0.01	<0.01	<0.01	<0.01	<0.01
	6	0.992	2	0.61	362	Obl 6 m	24.28	7.18	**0.59**	10.67		<0.01	**1.00**	<0.01	<0.01
	9	4.178	2	0.12	252	Obl 9 m	14.95	8.33	5.95	5.1	4.84		<0.01	<0.01	<0.01
Sheltered	3	5.175	2	0.08	1474	Shl 3 m	38.95	11.1	**1.15**	12.92	**0.25**	5.62		<0.01	<0.01
	6	5.453	2	0.07	619	Shl 6 m	45.43	24.25	12.72	22.29	11.12	15.17	15.67		0.02
	9	1.296	2	0.52	285	Shl 9 m	37.3	21.74	13.5	21.95	12.42	15.97	15.42	3.46	

**A)** No significant differences in drag forces were found among locations of equal depth and exposure. **B)** When sites were pooled by habitat and depth (exposed, oblique and sheltered habitats each at 3, 6 and 9 m depth), multiple comparisons revealed significant differences among all habitats, except the exposed 9 m, oblique 6 m and sheltered 3 m habitats (z-values on lower section, P-values on upper section in **bold**). FDR corrections were used to avoid Type I errors (α = 0.025) (Benjamini and Hochberg 1995).

## Results

### Current meter performance

Instrument calibration revealed that measures from 0–100% load had a precision of 0.055±0.044% and an accuracy of 0.513±0.653% (mean ±S.D.) with no detectable difference among individual instruments (Precision: F_8,53_ = 1.23, p = 0.31; Accuracy: F_8,53_ = 1.13, p = 0.36, Observed Power 0.80). Precision was also independent of applied load (F_5,53_ = 1.00, p = 0.43, Observed power 0.99) whilst accuracy increased from 1.285% to 0.046% as the applied load increased to 100% (F_5,53_ = 8.91, p<0.01). This increase in accuracy was caused by identical measuring increments from 0 to 100% load (10 bit resolution).

**Figure 3:**
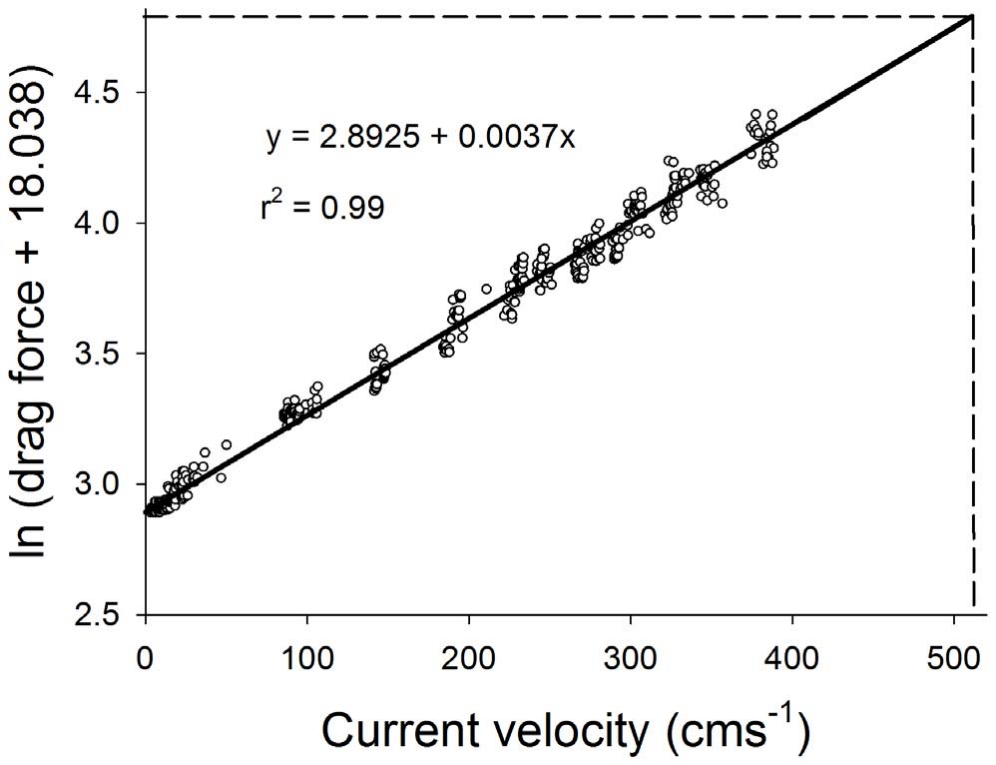
Correlation between drag forces (%) on the current meter sensor and water velocity (cms^−1^). Calibrations for low velocity (0–50 cms^−1^) were made by placing the current meter in a unidirectional current within a flume, whilst high velocity (>50–400 cms^−1^) was calibrated next to a Vernier LabQuest Flow rate sensor following the boat-tow methodology of Utter and Denny [28]. The log-linear correlation is highly significant (F = 12,090.7, p<0.0001).

Recorded values of current velocities (cms^−1^) displayed a significant log-linear correlation with drag forces (%) on the load cell sensors (Best fit: y = 2.8925 + 0.0037x, r^2^ = 0.99, See Fig. 3), and at current velocities between 0 and 400 cms^−1^ the instruments showed a measuring resolution of ±0.26 cms^−1^ (Fig. 3).

### Current velocity profile of a coral reef ecosystem on the Great Barrier Reef, Australia

**Figure 4:**
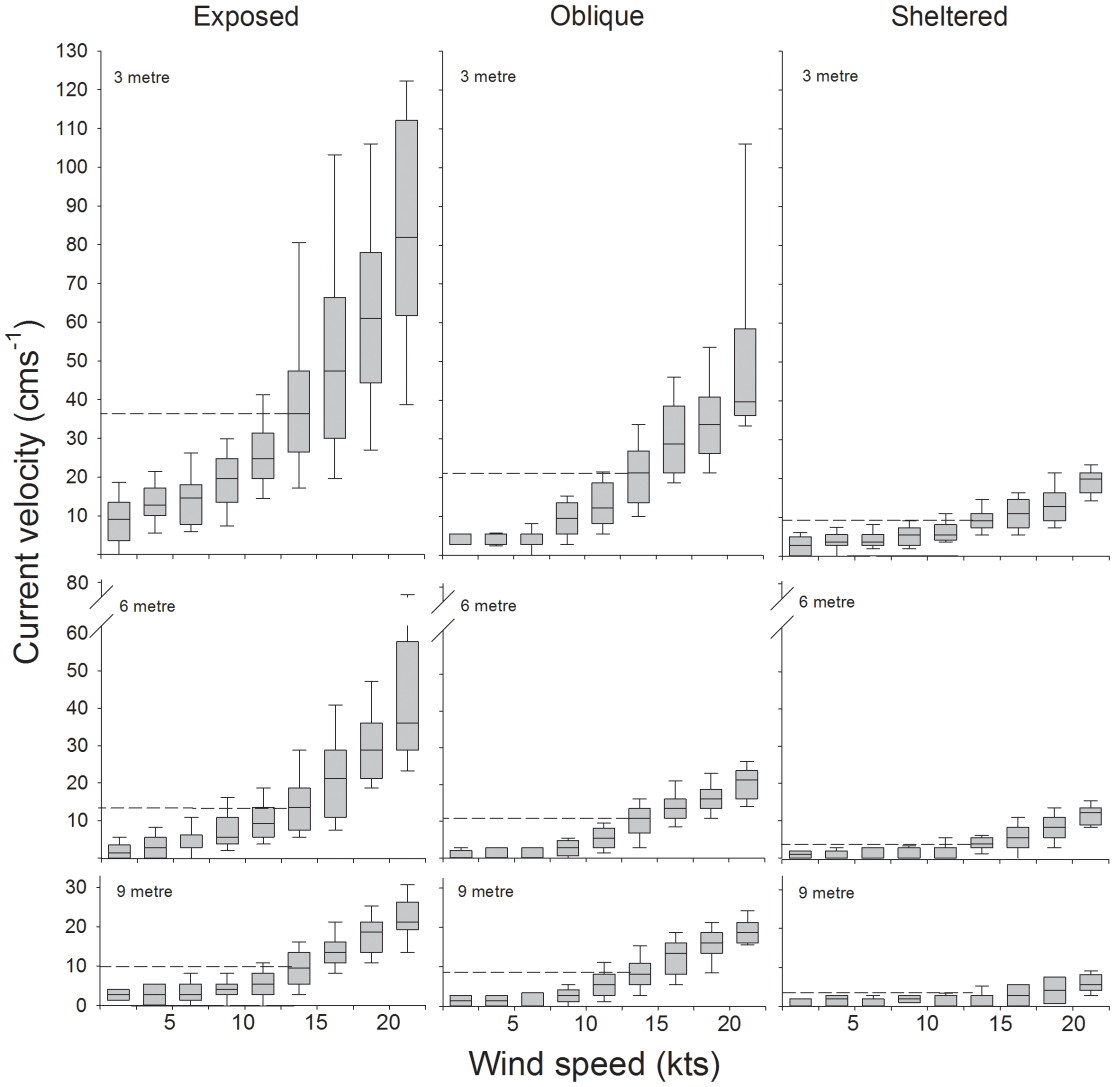
Current velocity profile of the coral reefs surrounding Lizard Island, Northern Great Barrier Reef, Australia. Maximum water velocities (cms^−1^) in exposed, oblique and sheltered habitats at 3, 6 and 9 m depth relative to a wind exposure of 0–22.5 knots hr^−1^ are shown. Boundary lines indicate the 25^th^ and 75^th^ percentiles, lines within the boxes represent median velocity values and the error bars indicate the 90^th^ and 10^th^ percentiles. Stippled lines indicate the most commonly encountered current velocity in each habitat relative to average daily wind velocities.

**Figure 5:**
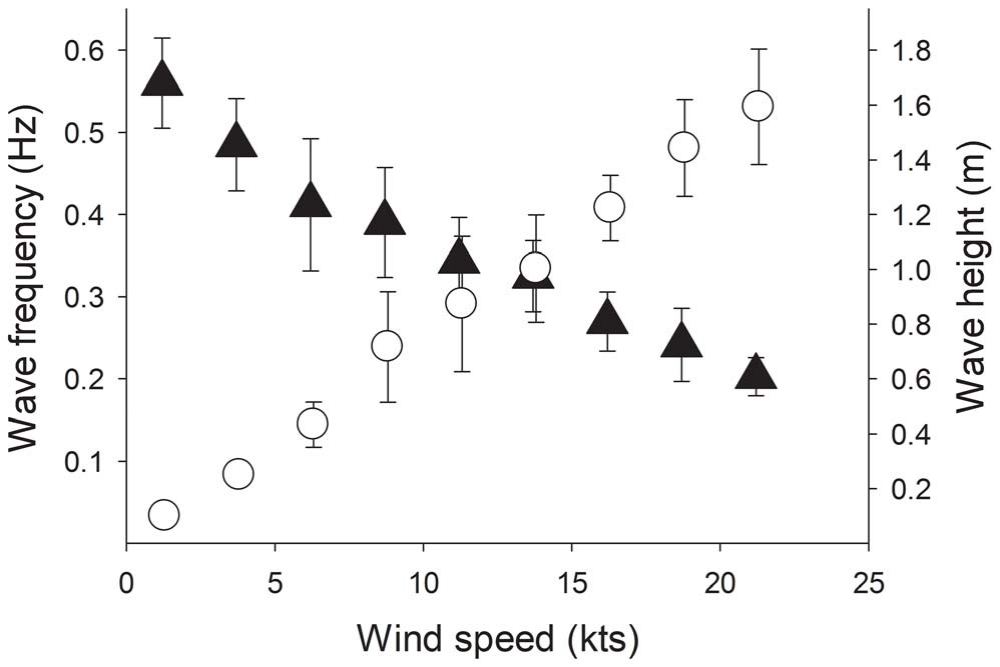
Wind induced wave frequency and amplitude in an exposed coral reef habitat (mean ±S.E.). Wave frequency (black markers) was measured on the exposed slope in front of Lizard Island, and wave amplitude (white markers) was estimated from marine forecasts for this location (www.bom.gov.au).

Current velocities across all examined habitats ranged between 0.8 and 82.0 cms^−1^ depending on habitat exposure, depth and wind velocity (Fig. 4). In general, habitats of different exposures and depths showed distinct and highly significant differences in current velocities (Kruskall-Wallis ANOVA, H_8_ = 3499.93, p<0.01, N = 7190). Only the exposed 9 m, oblique 6 m and sheltered 3 m habitats showed no significant differences in recorded velocities (Table 2). The highest current velocities were found at the crest of the exposed habitats (3 m depth, median: 9.2–82.0 cms^−1^) while the lowest velocity was found at 9 m depth in sheltered habitats (0.8–5.9 cms^−1^, Fig. 4). Across all habitats, current velocity increased exponentially with wind velocity and became increasingly more variable at the highest wind velocities (see Fig. 4, 5). Currents that coincided with periods of negligible wind (≤1 knots) revealed that tidal driven currents explained only 3.3±0.4 cms^−1^ (mean ±S.E.) of total water movement within both exposed and sheltered habitats (range 0–5.5 cms^−1^). Wave frequency reduced from 0.54±0.03 Hz to 0.20±0.02 Hz (mean ±S.E.) as wind velocity increased from 0 to 22.5knots, and estimated wave height increased to 1.6 m (Fig. 5).

## Discussion

This study uses load cell technology to measure water currents in aquatic ecosystems, at a resolution necessary for ecological studies on benthos and fish, and results notably illustrate the many advantages of this method in terms of automation, costs, data detail and acquisition flexibility in field environments.

The current meter instruments described here can register and process current velocity and wave movement at a frequency of 16 MHz and record the measures at >1 MHz, which covers the 0.6–1 MHz sampling frequencies often used for wave studies [35]–[36]. Low energy consumption allows data collection to run for several weeks continuously and the sensor can maintain a signal precision and accuracy of ≥99% for all current velocities. The instruments also offer 10 bit measuring resolution and a 98.1% correlation between sensor signal and current velocity to >400 cms^−1^. In particular, the maximum current velocities encountered in this study was equivalent to just 57.0% of the maximum drag force on the sensors, suggesting that these instruments are capable of measuring water velocity to >500 cms^−1^. Furthermore, the instrument housing is pressure rated to >194 psi theoretically allowing deployment to depths of 120 m, whilst the 0.5 mm zero stretch line leading to the load cell is capable of withstanding current or wave driven shear stresses on the drag sphere of >25 kNm^−2^. At less than $150 in component costs, these durable instruments provide affordable access to highly reliable velocity measures and allow simultaneous recordings of minimum, average and maximum current velocity and wave movement in multiple locations over time.

The field measures presented here also cover a significant gap in the current literature [15]–[16] by presenting a detailed profile of current velocities across depth and exposure gradients for a large section of a tropical coral reef system. Greater than 15-fold differences in maximum velocities were recorded among individual coral reef habitats (5.5 to >82 cms^−1^) and up to 7-fold differences among habitat sites that differ by only a few meters in depth. Increasing wind velocities from >0 to 22.5 knots caused up to 9-fold increases in current velocities within individual exposed habitats. Based on daily wind velocity data from this location between 2003–2011 [30], average yearly wind velocities are 12.6±0.3 knots with slightly stronger winds in winter (14.5±0.3 knots) and weaker winds in summer (10.8±0.3 knots, mean ±S.E.). Consequently, the most commonly occurring current velocities for these reefs range from 1.3 to 36.4 cms^−1^ depending on habitat exposure and depth (see Fig. 4).

Given the ease of instrument deployment and detail of data acquired, these instruments clearly provide a real-world alternative to existing methods for measuring currents in aquatic ecosystems (see e.g. [27]). These instruments may be useful for a wide variety of habitats and ecological questions. In rivers and streams with planar beds, for instance, these instruments could be used to measure the water velocity along different sections of the bed over prolonged timescales and demonstrate variations in current to different zones of the river and associated benthic community. Equally, continuous measures of currents in wave exposed coastal ecosystems and large lakes could clarify the physical forces impacting on resident species and help explain species compositions and community structures in individual habitats.

Results of this study allow performance measures of individual species to be compared to their particular habitat use and distributions patters. For instance, the current velocities ≥82 cms^−1^ recorded here had shear stresses exceeding 100 Nm^−2^ (calculated following *τ* = Force (N)/Area (m2)). Such forces arguably require benthic organisms to have tremendous structural strength, grip on the substrate and/or significant swimming abilities in order to survive. Given these conditions are measured on a mid-shelf reef which is relatively protected from direct open ocean swell, even harsher conditions may be expected in more exposed locations such as the outer Great Barrier Reef. Consequently, these results clearly highlight why the distribution and abundance of many reef species such as corals and teleosts has been linked to current velocities within individual habitats [19], [31], [37].

The presented instrument specifically measured currents over a 7 cm diameter drag-sphere and at a distance of 35 cm above the substratum befitting the size and location of many reef fishes swimming above the substratum [38]. However the scale of the instrument could easily be change to accommodate species that are closer or further from the substratum by extending or reducing the guide rod length (Fig. 1). Equally, a smaller combination of drag-sphere and load cell could be used for measuring current over smaller spatial scales, or the instrument could be inverted on a stand or buried to place the drag-sphere much closer to the substratum and thereby measure near bed velocities experienced by benthic organisms.

**Figure 6:**
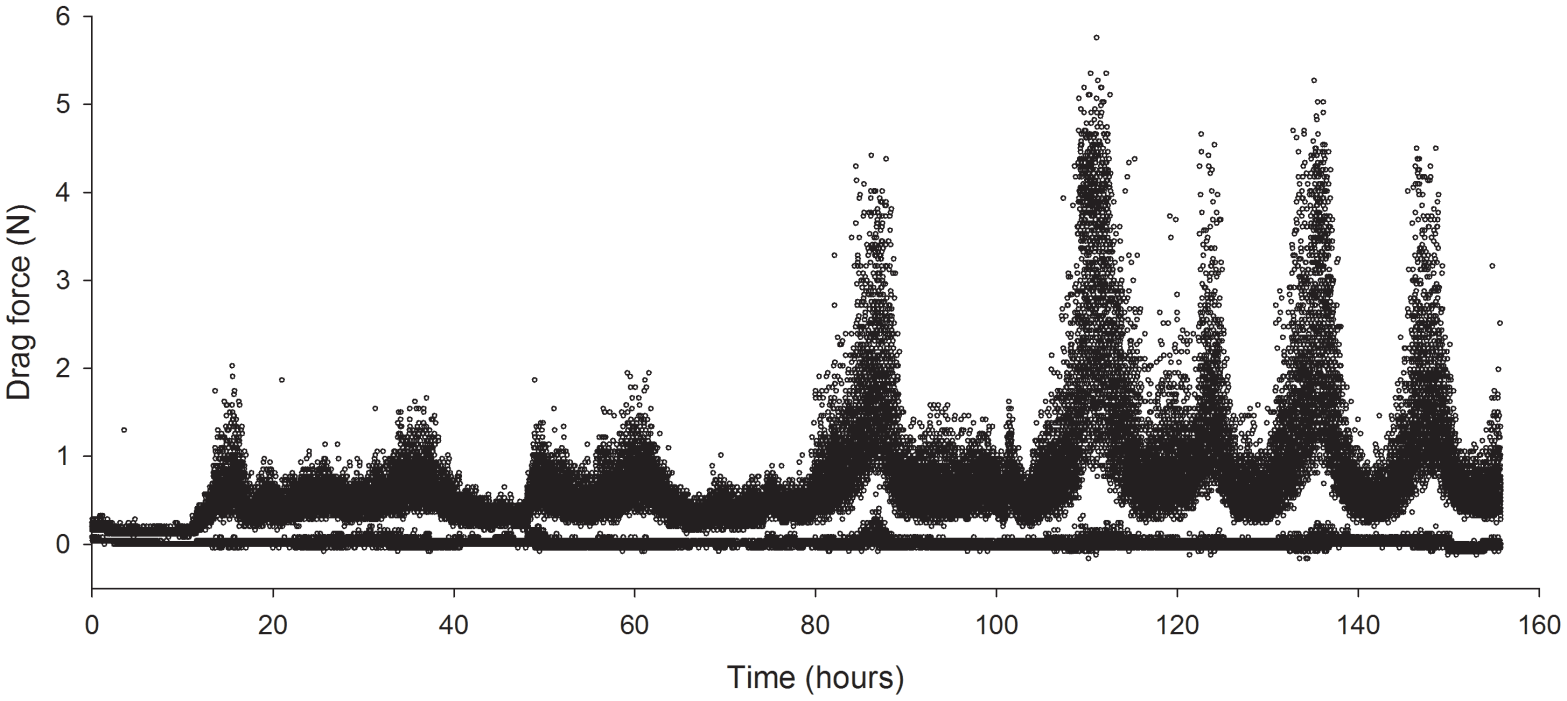
A raw data stream from a 7 day continuous measure of current velocity. Water velocity is measured as drag on the current meter drag-sphere and is here reported in Newton (N). In this data stream, drag forces were continuously measured at 16 MHz and for every 10 sec interval the single highest and single lowest measure were recorded. Notice how variation in drag forces increases with increasing current velocity. Notice also the stable minimum measures over time highlighting that no ascendible zero-drift occurred. The high measure (∼1.4 N) at the beginning of the data stream is a test pull made by the diver immediately after securing the current meter to the substratum, and indicates the start of data recording in the field location.

Using a programmable microcontroller allows for a high degree of freedom in deployment time and in the specific type and amount of data recorded. Once programmed, the Arduino Pro 328 used here can monitor time and date and specifically record data such as peak, minimum and average values (see example raw data trace in Fig. 6) which can be used to calculate water displacement and acceleration in addition to velocity. Importantly, this microcontroller can also be programmed to power down between measurements, thereby vastly reducing power consumption and significantly extending field deployment time to several months. Reduction in sampling frequency may equally lower power consumption and significantly extend deployment time.

Although current direction was not recorded in the present study, these data could be included by using a bi-directional or multi-directional load cell rather than the single point type described here. These cells measure forces in the x, y and z direction and would enable this instrument to measure the precise direction of water motion, whilst keeping power consumption at a minimum. Velocity time series during periods of steady conditions in e.g. stream beds could equally provide info on turbulence by calculating the standard deviation or root mean square of force measures. Importantly, measures of current direction, turbulence, or wave frequency significantly above the <1 Hz recorded here, may require an instrument housing and load cell sensor with greater structural stiffness to minimize instrument flex and maintain frequency response. However, such inclusions may substantially increase versatility for some users.

This instrument was deployed in an environment where shear stresses >100 Nm^−2^, emphasizing its durability under real-world harsh conditions. Whilst currents have previously been described for some tropical coral reefs (e.g. [5]), this is the first time a current velocity profile has been recorded for such a wide combination of habitats and weather conditions. Consequently, this instrument may significantly enhance our ability to link organism ecology with ambient currents in aquatic environments.

## Supporting Information

Figure S1 **A detailed schematic and assembly diagram of the instrument housing and sensor array.** Component descriptions and manufacturer can be seen in Table S1.
(TIF)

Table S1 **Flow meter component list.**
(DOCX)

Programming information S1 **An example of the programming used to control the flow meter instrument.**
(DOCX)
