# Supplementary figures and images for: Quantifying Water Flow within Aquatic Ecosystems Using Load Cell Sensors: A Profile of Currents Experienced by Coral Reef Organisms around Lizard Island, Great Barrier Reef, Australia
Source: PLoS One. 2014 Jan 8;9(1):e83240. doi: 10.1371/journal.pone.0083240 (PMC3885433; doi:10.1371/journal.pone.0083240)

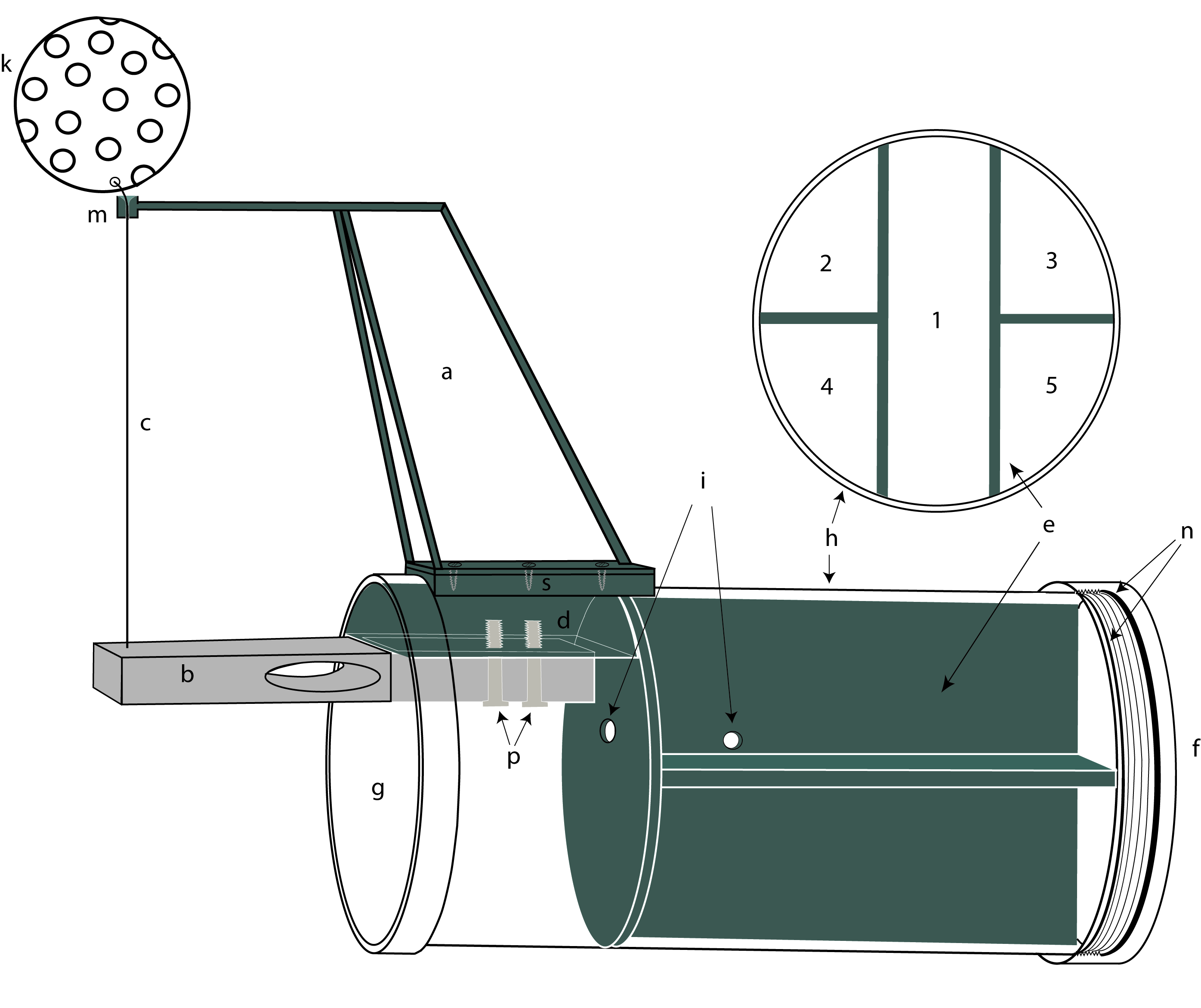

Supplement: Figure S1 — A detailed schematic and assembly diagram of the instrument housing and sensor array. Component descriptions and manufacturer can be seen in Table S1. (TIF) [file pone.0083240.s001.tif]
