# Supplementary material for: Quantifying Water Flow within Aquatic Ecosystems Using Load Cell Sensors: A Profile of Currents Experienced by Coral Reef Organisms around Lizard Island, Great Barrier Reef, Australia
Source: PLoS One. 2014 Jan 8;9(1):e83240. doi: 10.1371/journal.pone.0083240 (PMC3885433; doi:10.1371/journal.pone.0083240)
Supplement: Table S1 — Flow meter component list. (DOCX) [file pone.0083240.s002.docx]

**Supplemental Table S1. Flow meter component list.**

| **Reference**  **(Fig. S1)** | **Description** |
| --- | --- |
| **a** | Stainless steel frame which directs the zero stretch line from the load cell sensor to the drag sphere. The height of this frame determines the height above the substratum where flow measurements are conducted. It is made from 5mm diameter marine-grade stainless steel (316) and is secured to the instrument housing part “s” using screws. |
| **b** | Load cell sensor, model 628A, 3kg, purchased from [www.hzloadcell.com](http://www.hzloadcell.com). This sensor is screwed into the housing part “d”. |
| **c** | Zero stretch line, Platil Millennium Braid Line - 300m, 30lb, Grey, purchased from www.bcf.com.au. |
| **d** | Solid block of PVC welded to the inside of the instrument housing. This block is 30mm thick and 60mm long. |
| **e** | Solid strengthening walls made of 3mm thick PVC. These walls are welded in place and help stiffen the instrument housing as well as make compartments for the different electronic components (see 1-5 below). |
| **f** | Threaded access cap with internal O-ring seals. This cap is made of unplasticicised PVC (uPVC), part D109100 from www.iplex.com.au. |
| **g** | Solvent weld cap. This cap is made from 3mm thick uPVC, part D105100 from www.iplex.com.au |
| **h** | Instrument housing made of two sections of extruded uPVC welded together. The inner section is a pipe with an internal diameter of 110mm, wall thickness 3mm (part DSMH100). A male threaded access coupling is welded onto this inner pipe, outer diameter 116mm, wall thickness 3mm (part D058100). Total thickness of housing 6mm. Parts obtained from www.iplex.com.au. |
| **i** | Access holes between the inner compartments of the instrument housing, used for connecting wires. |
| **k** | Drag-sphere. This is a neutrally buoyant whiffle ball, 70mm diameter. |
| **m** | Guide rod with a conical internal access hole. This conical hole reduces friction from the zero stretch line attached to the drag-sphere. |
| **n** | Two 110mm O-ring seals made from EPDM polymer, used to make the instrument housing access cap water tight. Obtained from www.sealimports.com.au |
| **p** | Screws securing the load cell sensor to a solid block of uPVC (part “d”) |
| **s** | Solid piece of PVC welded onto the top of the instrument housing. This solid piece is used to stiffen the instrument housing and hold the stainless steel frame (part “a”) |
| **1-5** | These are internal compartments used to house the battery (1), signal conditioner (2), microcontroller and datalogger (3). The remaining two compartments (4 and 5) are filled with silica bags to absorb any build-up of humidity during deployment. |
| **solvent** | The uPVC components are all welded together using Iplex Solvent Cement Type P and Iplex Priming Fluid (www.iplex.com.au). |
