# Supplementary material for: Quantifying Water Flow within Aquatic Ecosystems Using Load Cell Sensors: A Profile of Currents Experienced by Coral Reef Organisms around Lizard Island, Great Barrier Reef, Australia
Source: PLoS One. 2014 Jan 8;9(1):e83240. doi: 10.1371/journal.pone.0083240 (PMC3885433; doi:10.1371/journal.pone.0083240)
Supplement: Programming information S1 — An example of the programming used to control the flow meter instrument. (DOCX) [file pone.0083240.s003.docx]

**Supplemental programming information: An example of programming used to control the flow meter instrument.** The following program is written in Arduino programming language (www.arduino.cc), which is a derivative of C++. Normal C++ programming would also work with the Arduino Pro 328 5 V / 16 MHz microcontroller and datalogger.

Program:

// Flow meter program V.1.5a. Created by Jacob Johansen, Feb 2011

// This version takes 1 measure every 0.1seconds and logs 1 signal to the SD card every 10 seconds.

// It also logs the following signals:

// One spot-measure recorded every 10sec,

// One minimum measure recorded as the lowest of the 99 preceding measures (i.e. 10 seconds),

// One average measure of the preceding 99 measures,

// One peak measure recorded as the highest of the preceding 99 measures (i.e. 10 seconds),

// the total number of signals recorded

// the total time passed since start

#include <Metro.h> // Include the Metro library

int interval = 100; // SET: interval for peak and minimum-measures (milliseconds)

Metro serialMetro = Metro(10000); // SET: the time period in between each stored measurement point in milliseconds

int measuringperiod = 10000; // SET: write the interval to the SD-Card

int ledPin = 13; // LED connected to digital pin 13

int analogValue = 0; // variable to hold the analog value from the Load Cell

int average = 0; // the average measure over the preceding 10seconds

int index = 0; // counter, number of samples for peak and minimum

int count = 0; // counter, number of samples for array

volatile int peakmeasure = 0; // Set peak measure to 0

volatile int minimum = 1023; // set minimum measure to 1023

long total = 0; // sum of all measures

unsigned long previousMillis = 0; // used for the interval between spot-measures

unsigned long previousmillis1 = 0;

unsigned long var = 0; // variable used by the counter

void setup() {

pinMode(ledPin, OUTPUT); // Set the led to output if needed for blinking later

Serial.begin(9600); // 9600bps is default for OpenLog

delay(1000); // Wait a second for OpenLog to init

Serial.println("Load Cell Signal program V.1.5a, Logger No 1 - ");

Serial.println("Created by Jacob Johansen, Feb 2011");

Serial.println(" ");

Serial.print("Logs are once every: ");

Serial.print(measuringperiod);

Serial.println(" milliseconds");

Serial.println(" ");

Serial.println("Note: Single spot, minimum, average and peak are all measured at 100Hz");

Serial.println(" ");

Serial.println("Values are: ");

Serial.print("Line No, Time since Start in milliseconds, Single Spot-measure, Minimum, Average, Peak, Number of signals used for calc"); }

void loop() {

analogValue = analogRead(0); // Read the analog input on pin 0:

unsigned long currentMillis = millis(); // Used for the interval between spot-measures

if (currentMillis - previousMillis >= interval) {

previousMillis = currentMillis; // Save the last time the last peak and min measure were taken

total = total + analogValue; // Add the analog reading to the total:

index = index + 1; // Advance to the next position in the array:

if (analogValue > peakmeasure) // If current measure is higher than peak, store in memory {

peakmeasure = analogValue ; // Store current analogValue in peak memory }

if (analogValue < minimum) // If current measure is lower than min, store in memory {

minimum = analogValue ; // Store current analogValue is minimum memory } }

if (serialMetro.check() == 1) // Check if the metro has passed it's interval. {

var++;

average = total / index; // Calculate the average measure:

Serial.println(" ");

Serial.print(var);

Serial.print(", ");

Serial.print(millis());

Serial.print(", ");

Serial.print(analogValue);

Serial.print(", ");

Serial.print(minimum);

Serial.print(", ");

Serial.print(average);

Serial.print(", ");

Serial.print(peakmeasure);

Serial.print(", ");

Serial.print(index);

Serial.print(", ");

index = 0; // Reset all variables

total = 0;

peakmeasure = 0;

minimum = 1023; }

count = 0; // Reset counter }
